# Case of Swallowing Artificial Teeth

**Published:** 1887-05

**Authors:** 


CASE OF SWALLOWING ARTIFICIAL TEETH.

M.D. writes: With reference to Mr. Ackery's letter, a patient of mine recently swallowed a plate (gold, with two teeth), and I immediately adopted a practice recommended to me some years ago by Sir James Paget in a similar case. I made him eat three good-sized slices of bread and swallow four tablespoonfuls of flour and water made into a fairly thick mass. I then administered an emetic, and the teeth returned entangled in the tenacious vomit. I may add that the first case was euually successful, and that something of this sort is habitually done at police-stations when prisoners passing false coins swallow them.—*British Medical Journal.*

